# The Roles of Environmental Factors in Regulation of Oxidative Stress in Plant

**DOI:** 10.1155/2019/9732325

**Published:** 2019-05-08

**Authors:** Xiulan Xie, Zhouqing He, Nifan Chen, Zizhong Tang, Qiang Wang, Yi Cai

**Affiliations:** ^1^School of Life Sciences, Sichuan Agricultural University, Ya'an 625014, China; ^2^Institute of Ecological Agriculture, Sichuan Agricultural University, Chengdu 611130, China

## Abstract

Exposure to a variety of environmental factors such as salinity, drought, metal toxicity, extreme temperature, air pollutants, ultraviolet-B (UV-B) radiation, pesticides, and pathogen infection leads to subject oxidative stress in plants, which in turn affects multiple biological processes via reactive oxygen species (ROS) generation. ROS include hydroxyl radicals, singlet oxygen, and hydrogen peroxide in the plant cells and activates signaling pathways leading to some changes of physiological, biochemical, and molecular mechanisms in cellular metabolism. Excessive ROS, however, cause oxidative stress, a state of imbalance between the production of ROS and the neutralization of free radicals by antioxidants, resulting in damage of cellular components including lipids, nucleic acids, metabolites, and proteins, which finally leads to the death of cells in plants. Thus, maintaining a physiological level of ROS is crucial for aerobic organisms, which relies on the combined operation of enzymatic and nonenzymatic antioxidants. In order to improve plants' tolerance towards the harsh environment, it is vital to reinforce the comprehension of oxidative stress and antioxidant systems. In this review, recent findings on the metabolism of ROS as well as the antioxidative defense machinery are briefly updated. The latest findings on differential regulation of antioxidants at multiple levels under adverse environment are also discussed here.

## 1. Introduction

The environment consists of a set of relationships between livings and nonliving things and is perfectly balanced by various natural processes. Each species influences its environment and, in turn, gets influenced by it. In general, numerous environmental factors including salinity, drought, extreme temperature, metal toxicity, air pollutants, ultraviolet light [1], and high doses of pesticides as well as pathogen infection can lead to subject oxidative stress in plants [2–6]. The oxidative stress is caused either by the direct effects of environmental stress or by indirect reactive oxygen species (ROS) generation and accumulation, which damage a cell before elimination. In order to evade stressors, animals are able to move and escape. Plants as sessile organisms, however, have developed complex strategies to release stressors. The plant cells will be in a state of “oxidative stress” if the ROS quantity is more than the inside defense mechanisms. It then exhibits growth retardation under oxidative stress, including flower and leaf abscission [7, 8], root gravitropism [9], seed germination [10], polar cell growth [11], lignin biosynthesis in cell wall [12], and cell senescence [13].

ROS include superoxide radical (O_2_^∙−^), hydroxyl radical (OH^*∙*^), hydrogen peroxide (H_2_O_2_), singlet oxygen (^1^O_2_), and so on [14, 15]. They are regarded as natural byproducts of the aerobic way of life and are generated in different cellular compartments like chloroplasts, peroxisomes, mitochondria, and plasma membrane [16]. It is significant that the increase of ROS level is highly reactive and affects a large variety of cellular, physiological, and biochemical functions, such as the disruption of plasma membrane via carbohydrate deoxidation, lipid peroxidation, protein denaturation, and the destruction of DNA, RNA, enzymes, and pigments [17–20]. All of those result in the loss of crop yield and quality [6, 21–27]. For example, in potatoes (*Solanum tuberosum L.*), overexpression of* AtCYP21-4*, a protein involved in oxidative stress tolerance, resulted in heavier tubers [28]. Similarly, in rice (*Oryza sativa L.*),* OsCYP21-4* overexpressing transgenic plants exhibited higher biomass and productivity with 10-15% higher seed weight than in the WT [28]. Besides, in sweet oranges (*Citrus sinensis L. Osbeck*), overexpression of* CitERF13* in citrus fruit peel resulted in rapid chlorophyll degradation and led to the accumulation of ROS [29, 30]. Moreover, in Arabidopsis (*Arabidopsis thaliana*), mutants of the singlet oxygen (^1^O_2_) overproducing flu and chlorina1 (ch1) have shown that ^1^O_2_-induced changes in gene expression can lead to either PCD or acclimation [31]. In conclusion, all of those observations demonstrate that ROS have a significant impact on crop yield and quality.

In the past several decades, research on oxidative stress was mainly focused on* Escherichia coli*. In the past ten years, however, it has moved beyond animals (e.g., human) to plants, particularly model plants and crops (e.g.,* Arabidopsis thaliana*, rice). It has substantially increased the understanding of the role and action of oxidative stress in general development-defense and environment-related responses [32–35]. Plants evolved their own antioxidant protection mechanism to maintain a dynamic balance of ROS, since the overcounteraction of ROS leads to the loss of an important intracellular signaling molecule [36].

This review primarily deals with the metabolism of ROS in plants and gives a brief introduction to the types, generation sites, and induced oxidative stresses of ROS. Then, we will focus on the antioxidative defense machinery in resisting the risk of overproduced ROS under disadvantageous environments and summarize recent researches on different environmental factors in regulating oxidative stress in plants.

## 2. The Metabolism of ROS in Plants

### 2.1. The Types of ROS

The most common ROS include O_2_^∙−^, ^1^O_2_, H_2_O_2_, and OH^*∙*^. The environment of molecular oxygen (O_2_) is generally inactive due to its electron configuration [37]. But the unbalanced metabolism of O_2_ can lead to the production of ROS, which include both free radicals (O_2_^∙−^, superoxide radical; OH^*∙*^, hydroxyl radical; HO_2_^∙^, perhydroxyl radical; and RO^*∙*^, alkoxy radicals) and nonradical molecules (H_2_O_2_, hydrogen peroxide; and ^1^O_2_, singlet oxygen) [5, 15, 38].

Among the various types of ROS, H_2_O_2_ received most attention. It plays a vital role in the regulation of senescence process [39], stomatal behavior [40], cell wall crosslinking [41], regulation of the cell cycle [42], photosynthesis [43], stress acclimation [44], and antioxidative defense [45]. In addition, it is indicated that H_2_O_2_ can interact with other signal molecules such as abscisic acid (ABA), auxin, brassinosteroid (BR), and ethylene, which are important for plant development and senescence [46–48]. Both ABA and BR induce heat and paraquat tolerance via H_2_O_2_ produced by* RBOH1* in tomato (*Solanum lycopersicum* L.) [49]. Moreover, ethylene mediates UV-B-induced stomatal closure through peroxidase-dependent H_2_O_2_ production in* Vicia faba* [50]. Besides, ethylene-induced stomatal closure is required for H_2_O_2_ synthesis, and both ethylene and H_2_O_2_ signaling mediate in guard cells in* Arabidopsis* [51]. The specificity of these responses allowing different signaling transduction pathways to act according to surrounding environmental triggers perceived and the physiological status of the plants is likely to be determined by spatial-temporal changes in H_2_O_2_ production and accumulation.

Several recent studies have demonstrated that H_2_O_2_ is involved in stress signal transduction pathways, which can activate multiple acclamatory responses that reinforce resistance to various biotic and abiotic stressors. Overexpression of pepper (*Capsicum annuum*)* CaWRKY41* in* Arabidopsis* indicated that it impaired Cd tolerance, enhanced Cd levels through activating Zn transporters, and accelerated H_2_O_2_ accumulation. On the contrary,* CaWRKY41 *silenced via VIGS in pepper plants displayed increased Cd tolerance and reduced H_2_O_2_ levels [52]. Mutations of* Cu/Zn-SOD1* (*csd1*),* csd2,* and* sodx* led to enhanced resistance to* Magnaporthe oryzae* and increased H_2_O_2_ accumulation in rice. Further studies revealed that they altered the expression of CSDs and other SOD family members, resulting in increased total SOD enzyme activity and leading to higher H_2_O_2_ production compared to WT [53]. These transgenic studies established the role of H_2_O_2_ in the formation of plant tolerance to different biotic and abiotic stresses.

### 2.2. The Production Sites of ROS

ROS are generated in both unstressed and stressed plant cells. Gradual reduction of O_2_ by high-energy exposure or electron-transfer reactions leads to the production of highly reactive ROS. In plants, the activation of ROS is energy dependent and requires an unavoidable leakage of electron from the electron transport activities of chloroplasts, peroxisomes, mitochondria, plasma membranes, endoplasmic reticulum (ER), apoplasts, and cell wall or as a byproduct of various metabolic pathways localized in different cellular compartments [48, 54–59].

Chloroplasts and peroxisomes are the main ROS generators in the presence of light, whilst the mitochondria are the chief sources of ROS production under dark conditions [1]. The chloroplast consists of a highly ordered system of thylakoids, which harbors the efficient light-capturing photosynthetic machinery. Photosystem (PS) I and PSII form the core of the light-harvesting systems in the thylakoids and are the primary sources of ROS generation [60, 61]. Near the reaction centers of PSII, O_2_ may produce ^1^O_2_ when there is overexcitation of chlorophyll under stress conditions. Besides, O_2_^∙−^ may also be formed at PSI via Mehler reaction [62] or at PSII during electron transfer to O_2_ through Q_A_ and Q_B_ [55]. Additionally, due to the activities of flavin oxidases, peroxisomes are the main sites of H_2_O_2_ generation [58, 63]. In mitochondria, O_2_^∙−^ and H_2_O_2_ may be generated by univalent reduction of O_2_ near electron transport chain in plant cell [57].

Apart from those organelles, there are cellular sites mediated in the generation of ROS. At plasma membrane that plays a vital role in sensing environmental conditions, localized NADPH-dependent oxidase transfers electrons from NADPH on cytoplasmic side to O_2_ producing O_2_^∙−^ [59]. ER also mediates the generation of O_2_^∙−^ by Cyt P_450_ [64]. During harsh environmental conditions, the apoplast is rendered for H_2_O_2_ production by stress signals combined with ABA [65]. As cell wall localized peroxidase(s), diamine/polyamine oxidases and oxalate oxidase produce H_2_O_2_ that may, in turn, be metabolized to OH^*∙*^ by the activity of class III peroxidases [66, 67].

### 2.3. Oxidative Damage

When the level of ROS is low or moderate, they function as second messenger that mediates a series of reactions in plant cells, including stomatal closure, programmed cell death (PCD) [23], gravitropism [81], and acquisition of tolerance to both abiotic and biotic stresses [82]. However, in the past two decades, it has become more and more evident that all types of ROS at a high concentration are significantly harmful to organisms. Constant environmental stresses for plants will lead to the generation of superfluous ROS which cannot be completely disposed by the active oxygen scavenging system. Therefore, important physiological actions should be exerted, such as peroxidation of lipids, oxidation of nucleic acids, denaturation of proteins, inhibition of enzyme activity, and even activation of PCD pathway [55, 59].

The major targets of oxidative damage caused by ROS are lipids and proteins in plant cell. The oxidative decomposition of polyunsaturated lipids in plasma membrane, which is known as lipid peroxidation, occurs in every organism and is often considered as an indicator to determine the extent of lipid damage under severe conditions [83–85]. It is now well demonstrated that lipid peroxidation starts a reaction chain that can also create other reactive products such as ketones, aldehydes, and hydroxyl acids and can modify proteins, by oxidation of some amino acid residues [86, 87]. The activity of the protein is altered due to modifications such as glutathionylation, carbonylation, nitrosylation, and disulfide bond formation [88].

## 3. Antioxidative Defense System in Plants

Environmental factors such as salinity, drought, chilling, metal toxicity, air pollutants, UV-B radiation, and high doses of pesticides as well as pathogen infection lead to enhanced production of ROS in plant cells [89, 90]. Plenty of studies demonstrated the significance of intracellular antioxidant defense machinery against a variety of stresses [91–93]. This antioxidant defense machinery includes enzymatic and nonenzymatic components to scavenge ROS, and it operates at different subcellular compartments such as chloroplasts, peroxisomes, plasma membranes, and ER [59]. Enzymatic antioxidants contain enzymes such as superoxide dismutase (SOD), catalase [84], guaiacol peroxidase (GPX), ascorbate peroxidase (APX), guaiacol peroxidase (GPOX), monodehydroascorbate reductase (MDHAR), dehydroascorbate reductase (DHAR), glutathione reductase (GR), and glutathione S-transferases (GST) and nonenzymatic antioxidants which are ascorbic acid, glutathione, carotenoids, tocopherols, proline, glycine betaine, and flavonoids [94]. Additionally, NADPH oxidases and respiratory burst oxidase homologues (RBOHs) are also known to be major components of ROS production system in plants [95].

Initially, most of the studies on antioxidative defense system were focused on enzymatic characteristics due to the limitations of the experimental conditions. The enzymes of SOD, APX, CAT, etc. have been widely investigated in order to understand the antioxidative defense mechanisms in response to oxidative stress induced by various environmental factors. For instance, in alfalfa (*Medicago sativa* L.), after NaCl treatment, Xinmu No. 1 exhibited higher enzymatic activity of SOD, APX, and CAT in its shoots and roots than Northstar and, meanwhile, showed lower levels of H_2_O_2_ production and lipid peroxidation [96]. In another example, blue light illumination increased fruit color index, enhanced the activities of SOD, CAT, and APX, and maintained lower levels of H_2_O_2_ in strawberry (*Fragaria vesca*), which demonstrated that the treatment of blue light maintains fruit quality and increases nutritional value in strawberries due to the strengthening of both antioxidant systems and free radical elimination capabilities [97].

Subsequently, with the development of molecular cloning technology, researches on the functions of antioxidant genes generated many new insights into this area. The dynamic transcription activity of ROS-scavenging enzymatic genes has been widely characterized. In pear (*Pyrus communis* L.), the expression of* SOD*,* CAT,* and* APX* were significantly upregulated over 24, 48, 72, and 96 h after inoculation of* Erwinia amylovora*, comparing to the controls [98]. In cotton (*Gossypium hirsutum*), the expression patterns of 18* GhSOD* genes were tested in different abiotic stresses, which indicated that they may play a very crucial role in ROS scavenging caused by various stresses through genome-wide characterization [99]. These expression patterns of* SOD*,* CAT,* and* APX* in pear and* GhSOD *suggest that they are associated with the antioxidative defense process. There have been a large number of similar studies on antioxidant genes expression in plants, focused mainly on the mRNA level, but further functional studies are limited.

In recent years, numbers of transgenic plants such as* Arabidopsis*, tomato, rice, tobacco, and maize have been developed with disposed expression of antioxidant enzymes that exhibited increased tolerance to salinity, extreme temperatures, and drought stress [100]. Jing et al. reported that overexpressing* Kandelia candel KcCSD* (a Cu/Zn SOD) in tobacco showed salinity tolerance in the aspect of lipid peroxidation, root growth, and survival rate and enhanced SOD and CAT activity compared to wild type (WT)[68]. Likewise, overexpression of Chinese cabbage (*Brassica campestris*)* BcAPX2* and* BcAPX3* in* Arabidopsis* improved seed germination rate and showed amazing high temperature tolerance via efficient scavenging of cellular H_2_O_2_ [73]. In* Arachis hypogaea*, transgenic* AhCuZnSOD* in tobacco plants resulted in enhanced salinity and drought tolerance as indicated by better seed germination and higher chlorophyll content compared to WT [69]. Notably, overexpression of a single gene could increase plant tolerance to different stresses and many researchers paid close attention to transgenics with overexpression of* SOD* for enhancing stress tolerance [90].

As science advances, a growing amount of researches show that the stress tolerance can develop markedly by applying the simultaneous coexpression of genes involved in metabolic pathways. Xu et al. (2014) coexpressed* MeCu/ZnSOD* and* MeAPX2* in cassava (*Manihot esculenta* Crantz) and tested the tolerance of transgenic plants against oxidative and chilling stresses. After exposure to 100 *μ*M methyl viologen and 0.5 M H_2_O_2_, the result exhibited a lower level of chlorophyll degreening, lipid peroxidation, and H_2_O_2_ accumulation along with a higher level of activities of SOD and APX in transgenic plants than the WT [75]. Similarly, coexpression of* Brassica rapa BrMDHAR* and* BrDHAR* genes via hybridization conferred tolerance to freezing [101]; cotransformation of* cytSOD* and* cytAPX* led to salinity tolerance in transgenic plums [102]; coexpression of* PaSOD* and* RaAPX* genes from* Potentilla atrosanguinea* and* Rheum austral,* respectively, in transgenic* Arabidopsis *showed increased salt tolerance through regulating lignin deposition [70].

Genes encoding enzymes required for antioxidative defense have been widely studied in several types of plants. However, research on the transcriptional regulation of antioxidant enzymes remains limited and mainly focuses on the oxidative stress-related transcription factors including AP2/ERF, NAC, MYB, and bHLH family [15, 103–105]. For example, overexpression of the buckwheat (*Fagopyrum tataricum*)* FtbHLH3* in* Arabidopsis *resulted in enhanced drought tolerance, which was attributed to not only the lower level of H_2_O_2_ but also the higher activities of SOD and CAT as well as the higher photosynthetic efficiency in transgenic lines compared to WT [106]. Overexpressing of rice miR529a led to enhanced plant resistance to high level of H_2_O_2_, which manifested as improved seed germination rate and increased SOD and POD activities, as well as reduced leaf rolling rate and chlorophyll content [107]. However, the underlying regulatory mechanisms specific to antioxidant enzymes are still not fully characterized and should be further explored.

## 4. The Impact of Environmental Factors on Plant Oxidative Stress

### 4.1. Salinity

Soil salinity is a major issue that limits the productivity and quality of the agricultural crops in many arid and semiarid regions of the world. Hypersaline conditions impact the stressed crops at multiple aspects such as oxidative stress, genotoxicity, ionic imbalance and toxicity, nutrition deficiency, and osmotic stress, resulting in subhealthy status of the plants [108]. As a consequence, plant cells decrease photosynthetic electron transport and generate excessive ROS. To counteract the deleterious effects mentioned above, plants have developed various strategies, including salt compartmentalization and exclusion [109].

In plants, all enzymatic scavengers operate together to conquer salt stress for better growth and development. In maize seedlings organs including roots, mature leaves, and young leaves, the activities of CAT and DHAR increased in all organs of salt-treated plants, while SOD, APX, GST, and GR increased specifically in the roots after NaCl treatment [110]. Two local wheat salt-tolerant cultivars, BARI Gom 27 and 28, displayed reduced accumulations of H_2_O_2_ and higher activities of CAT, peroxidase, and APX than salt-sensitive cultivars in virtue of reduced oxidative damage [111]. In the above reports, higher expression level of enzymatic antioxidants induced by salt treatment suggests an efficient way to decrease saline toxicities. However, some studies also indicated that differential expression behavior of these enzyme genes, the salinity extent, and the exposure time as well as the plant developmental stage will make the expression levels different [53, 71, 72].

Due to the significance of antioxidant enzymes, genetic engineering with altered antioxidant entities through overexpression of their pathway genes has been conducted to improve salt tolerance in various crops [72]. Zhou et al. (2018) demonstrated that Tyr-210 is a major phosphorylation site in CatC and is activated by STRK1 (receptor-like cytoplasmic kinase). Moreover, phosphorylating and activating CatC by overexpressing STRK1 regulated H_2_O_2_ homeostasis and indeed improved salt and oxidative tolerance. Importantly, overexpression of* STRK1* in rice enhanced rice seedling growth status; meanwhile, the loss of grain yield under salt stress was significantly limited [112]. Guan et al. (2015) found that the expression of* PutAPX* was upregulated with extended exposure to NaHCO_3_, NaCl, H_2_O_2_, and PEG6000 treatment in* Puccinellia tenuiflora*. Furthermore, when grown with 150 or 175 mM NaCl, transgenic Arabidopsis plants overexpressing* PutAPX *displayed increased tolerance of saline toxicity and decreased level of lipid peroxidation [71].

### 4.2. Drought

Drought is an important environmental stress for plant growth that ultimately causes the reduction in crops yield in a global warming world, especially for commercial crops including rice, wheat, and maize [113]. Nevertheless, plants have evolved multiple strategies to minimize the damage during drought conditions [108]. It is demonstrated that the key process in plant physiological response to drought is the production of ROS, which causes progressive oxidative damage, stunted growth, and eventual cell death when ROS level reaches a certain threshold [89].

Several researches indicated that sustainable tolerance to drought stress could be achieved by increasing the expression/activity of ROS scavenging-related genes/enzymes [96]. For example, overexpression (OE) of* OsLG3* (a ERF family transcription factor) increased rice drought tolerance by modulating ROS homeostasis through upregulation in the OE lines and downregulation in the RNAi lines of the expression of 10 ROS scavenging-related genes (*APX1*,* APX2*,* APX4*,* APX6*,* APX8*,* CATB*,* POD1*,* POD2*,* SODcc1*, and* FeSOD*) [114]. Moreover, Xu et al. (2016) reported that the increasing cytokinin production through overexpression of* isopentenyl transferase* (*ipt*) alleviated drought damage and promoted root growth in* Agrostis stolonifera*. Further enzymatic assays and transcript abundance analysis showed that CAT, SOD, POD, and DHAR were much higher in roots of a transgenic line overexpressing* ipt* under drought stress [74]. In another example,* Arabidopsis *ZAT18 (a C2H2 zinc finger protein) OE plants exhibited less leaf water loss, lower content of H_2_O_2_, higher leaf water content, and higher activities of POD and CAT after drought treatment when compared with the WT [115].

The significant roles of oxidant enzymes in ROS scavenging also have been suggested by studies with transgenic plants. Wang et al. (2005) demonstrated that overexpression of pea (*Pisum sativum*)* MnSOD* in rice showed reduced electrolyte leakage compared to WT leaf slices after polyethylene glycol 6000 treatment, which could induce drought stress [116]. Lu et al. (2010) reported that overexpressing* APX *and* Cu/ZnSOD* in chloroplasts of sweet potato improved the capacity of drought tolerance and recovery in plants. It also exhibited enhanced photosynthetic activity when suffered drought stress, compared to WT [117].

### 4.3. Chilling

Chilling stress is a major restriction of crops growth, production, and distribution. Enhancing crop chilling tolerance is thus vital to crops yield increase. As chilling induces oxidative stress and results in lipid peroxidation, chlorophyll degradation, etc., chilling tolerance is thus mainly associated with antioxidant enzyme activities enhancement and corresponding H_2_O_2_ accumulation reduction (Table 1).

Glutaredoxins (GRXs), as common oxidoreductases, mainly utilize the reducing power of glutathione to break disulfide bonds of substrate proteins and maintain cellular redox homeostasis. It has been reported that the expression of* AtGRXS17* in tomato conferred transgenic tomato chilling stress tolerance without any growth defects showing up. Compared with wild-type plants, tomato expressing* AtGRXS17* exhibits lower ion leakage and increased maximal photochemical efficiency when challenged by cold [76]. Soluble sugar in those transgenic tomato plants also accumulates to a higher level.

Xu et al. (2014) coexpressed* MeCu/ZnSOD* and* MeAPX2* in cassava (*Manihot esculenta* Crantz) to enhance tolerance against oxidative attributed to chilling stresses. Specifically, higher levels of antioxidative enzymes activities and lower levels of chlorophyll degradation, lipid peroxidation, and H_2_O_2_ accumulation were detected in transgenic plants after exposure to H_2_O_2_ and methyl viologen, a ROS-generating reagent [75]. Similarly,* BrMDHAR* and* BrDHAR* coexpression in* Brassica rapa* via hybridization elevated the plant resistance to freezing [101].

### 4.4. Metal Toxicity

Since the industrial revolution, heavy metal environmental pollution has become so serious that an increasing number of scientists are engaged in relevant scientific research. Usually, the concentrations of heavy metals determine their negative impacts on plants and the environment [118]. Plants then exhibit their ability to avoid the detrimental impacts when the amount of heavy metals is controlled in a natural level [119]. There has already been evidence suggesting that excessive level of heavy metals impairs homeostasis and increases ROS production in the plant cells [120].

Due to the redox ability, heavy metals absorbed by plants are involved in several mechanisms that produce free radicals. As redox-active elements, iron (Fe), copper (Cu), chromium [121], etc., can participate in a redox-cycling reaction, resulting in the production of toxic hydroxyl radicals which seriously damage the living cells. Mannitol exhibits the ability to activate the antioxidant enzyme which might be helpful to alleviating pathological symptoms in wheat (*Triticum aestivum* L.) when challenged by Cr stress [122]. As for other metals without redox capacity, such as lead (Li), cadmium (Cd), mercury (Hg), zinc (Zn), and nickel (Ni), the primary route for their toxicity is to suppress the antioxidative system, which can be achieved by depleting glutathione and binding sulfhydryl groups of antioxidative enzymes including reductases, superoxide dismutase, and catalases [77]. They also meddle with photosynthetic process and consequently increase the superoxide and singlet oxygen generation within the cells [123]. As highlighted by several authors, the intensity of oxidative stress induced by heavy metals depends on species and varies across disparate genotypes, tissues, and/or developmental stages. In general, metal-susceptible plants display marked symptoms under oxidative stress, while metal-resistant plants display only mild or even no oxidative damages [119].

In addition to the antioxidant responses in plants, there have been a number of chemicals reported that may reduce the uptake of heavy metals and ameliorate the oxidative stress in plants. For example, the biochar derived from* Citrus epicarp* inhibited* Abelmoschus esculentus *(L.) Moench (okra) to absorb Cd from low Cd stress environment [124]. Decreasing Cu uptake and oxidative damage through applying exogenous SNP (Sodium Nitroprusside) and GSH (Glutathione) also alleviated copper toxicity in rice seedlings [78]. Besides, some fungus can provide plants with protections via mycorrhization [125].

### 4.5. UV-B Radiations

UV-B radiation (280 - 315 nm) accompanies exposure to sunlight and is an inevitable abiotic factor for photosynthetic organisms. When plants are exposed to high level of UV-B radiation, a plethora of cell components, particularly the cellular macromolecules (DNA and protein), are interfered, and oxygen radicals are induced as a consequence. The effects of these radiations vary from the applied dose and sensitivity of living plant cells to the action of radiation type [126].

It has been known for many years that exposure of crop plants to physical radiations such as ionizing FN and nonionizing UV-B generates excessive free radicals which give rise to cytogenetic changes in plants [79]. A current study observed that almost all irradiation exposure doses of FN and UV-B exhibited special interference with meiotic-pollen mother cells and pollen grains leading to the genotoxic effect in* Vicia faba* L. [127]. The RUS1/RUS2 (Root UV-B Sensitive) complex, which works in UV-B-sensing pathway in root, is involved in seedling morphogenesis and development at early stages in Arabidopsis. In the absence of RUS1/RUS2 complex, the development of seedling is interfered with due to the dramatically increased signal generated from photoreceptors after the perception of UV-B [128].

### 4.6. Pathogens

Pathogen infection which causes plant diseases and epidemics has threatened plant growth, crop yield, and food security worldwide. Diverse and rapidly evolving characters make pathogen one of the most disastrous threats for plants. Different from vertebrates, sessile plants developed a conserved, unique yet sophisticated immune system to combat invading pathogens. Physical and chemical barriers deal with most of the microbes, while specific resistance responses termed host resistance handle the rest of them. When plants perceive PAMPs (Pathogen-associated Molecular Patterns), a multitude of immune responses within the cells will be triggered [129, 130].

ROS production is one of the responses mentioned above that bursts rapidly and transiently. It is mediated by NADPH oxidases located in plasma membrane, belonging to the respiratory burst oxidase homolog (RBOH) family [131–133], as well as apoplastic peroxidases (PRXs). The mechanism of RBOH in stress response is well studied [134]. Kadota* et al. *(2014) has demonstrated that RBOHD phosphorylation mediated by BIK1 has biological significance for stomatal closure, ROS burst, and disease resistance against bacterial pathogens in PTI (PAMP-triggered immunity) [95]. Another example of ROS-related defense response is regulated by ACD11, whose binding partners are Arabidopsis BPA1 and its homologs. Those binding partners can be targeted by an effector, RxLR207, derived from* Phytophthora capsici.*, and then ROS-mediated cell death, which is indispensable for virulence of* P. capsici.*, is activated [135].

Another way to enhance plant disease resistance is to prevent H_2_O_2_ degradation catalyzed by peroxidases and to increase ROS burst to eliminate invading pathogens. A recent discovery found a natural mutation of the transcription factor that suppresses peroxidase expression and confers broad-spectrum blast resistance in rice [80].

Nevertheless, the transcriptional regulation of ROS-related genes in the apoplast remains largely controversial. For example, the expression of the apoplastic peroxidases coding genes* PRX33* and* PRX34* enhanced cytokinin-mediated stomatal immunity and plant resistance to bacteria [136]. It has also been reported that plants knocked down* PRX33* and* PRX34* exhibited enhanced disease resistance to the necrotrophic fungus* Alternaria brassicicola* [137].

Although ROS burst and accumulation cause damage to plant cells, the generation of ROS is indispensable in plant immunity. Owing to the signaling and bactericidal functions of ROS, short-term oxidative stress is utilized by plant immune system as an effective way to defend against pathogens. The dual roles of ROS in signal transduction help plants detect pathogen invasion. Still, the production of ROS needs to be strictly regulated to control its function.

ROS and Ca ^2+^ waves contribute to rapid systemic signaling, which is crucial to plant adaptation to abiotic stresses [138]. Besides, plant resistance to pathogens can be attenuated or enhanced by abiotic stress factors [139]. ROS production bursts in a few minutes after immunogenic treatment; thus, this biological process can be detected to uncover the contribution of those plant components that are necessary in the burst of early immune responses [133].

## 5. Conclusions

**Figure 1:**
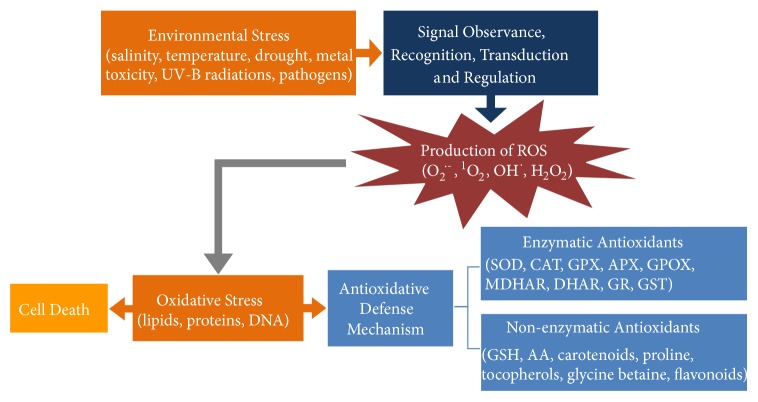
Environmental stress induced ROS generation, antioxidative defense, and cell death in plant.

Plants face a variety of pressure during their growth, especially environmental pressure including salinity, drought, extreme temperature, metal toxicity, UV-B radiation, pesticides, and pathogen infection. They adapt to those conditions with adjustments at molecular, biochemical, and physiological levels, especially via antioxidant systems. Although the general process of ROS formation as well as the antioxidative defense of plants (Figure 1) are understood, it is still unclear how plants detect stresses and prepare themselves for the incoming threats because of the extremely reactive nature and short half-life of ROS. In recent studies, genetically modified plants with overexpressing functional genes have shown promising traits in combating oxidative stress. Moreover, to achieve high tolerance against various adverse environments, efforts should be made to generate transgenic plants by coexpressing multiple effective genes.

## Figures and Tables

**Table 1:** Antioxidant enzymatic defense mechanism in response to oxidative stress induced by various environmental factors.

Environmental factors	Antioxidant enzymes	Plant species/source crop	Recipient crop	References
salinity	Cu/ZnSOD, CAT	*Kandelia candel*	*tobacco*	[68]
	SOD	*Arachis hypogaea*		[69]
	PaSOD, RaAPX	*Potentilla atrosanguinea Rheum austral*	*Arabidopsis*	[70]
	PutAPX	*Puccinellia tenuiflora*	*Arabidopsis*	[71]
	OsAPX	*Oryza sativa*	*knockout*	[72]
drought	APX	*Solanum melongena*	*Oryza sativa*	[73]
				[74]
chilling	SOD, APX	*Manihot esculenta*		[75]
	Glutaredoxins	*Arabidopsis thaliana*	*Solanum lycopersicum*	[76]
metal toxicity	GR	*Cannabis sativa*	*Cannabis sativa*	[77]
	GSH	synthetic	*Oryza sativa*	[78]
UV-B radiations	APX, SOD, POD, CAT	*Pisum sativum Cassia auriculata*		[79]
pathogens	peroxidase expression	*Oryza sativa*	mutation	[80]
